# Intelligent virtual agents as language trainers facilitate multilingualism

**DOI:** 10.3389/fpsyg.2014.00295

**Published:** 2014-04-14

**Authors:** Manuela Macedonia, Iris Groher, Friedrich Roithmayr

**Affiliations:** ^1^Department of Information Engineering, Johannes Kepler UniversityLinz, Austria; ^2^Max Planck Institute for Human Cognitive and Brain Sciences, Research Group Neural Mechanisms of Human CommunicationLeipzig, Germany

**Keywords:** intelligent virtual agent, multilingualism, enactment effect, memory enhancement, mobile device

## Abstract

In this paper we introduce a new generation of language trainers: intelligent virtual agents (IVAs) with human appearance and the capability to teach foreign language vocabulary. We report results from studies that we have conducted with Billie, an IVA employed as a vocabulary trainer, as well as research findings on the acceptance of the agent as a trainer by adults and children. The results show that Billie can train humans as well as a human teacher can and that both adults and children accept the IVA as a trainer. The advantages of IVAs are multiple. First, their teaching methods can be based on neuropsychological research findings concerning memory and learning practice. Second, virtual teachers can provide individualized training. Third, they coach users during training, are always supportive, and motivate learners to train. Fourth, agents will reside in the user's mobile devices and thus be at the user's disposal everywhere and anytime. Agents in apps will make foreign language training accessible to anybody at low cost. This will enable people around the world, including physically, financially, and geographically disadvantaged persons, to learn a foreign language and help to facilitate multilingualism.

Everybody knows how tedious learning a foreign language can be, not only in school. Many of us have already quit a class in adult education because the teaching was inefficient or we did not study enough at home, or simply because we could not manage to get to the lessons on time. Nowadays more than ever, high proficiency in the world's major languages such as English and Spanish has become a must. However, formal instruction alone cannot provide adequate training for everybody. As a result, many students who graduate from high school cannot speak a lingua franca fluently enough to interact in business or science, or simply to acquire information from international media. In the future, intelligent virtual agents (IVAs) could provide what learners lack in formal instruction. Rigorous assessment of the effects of IVAs on learning can facilitate their introduction into learning environments.

## Virtual agents can already train humans on vocabulary

**Figure 1:**
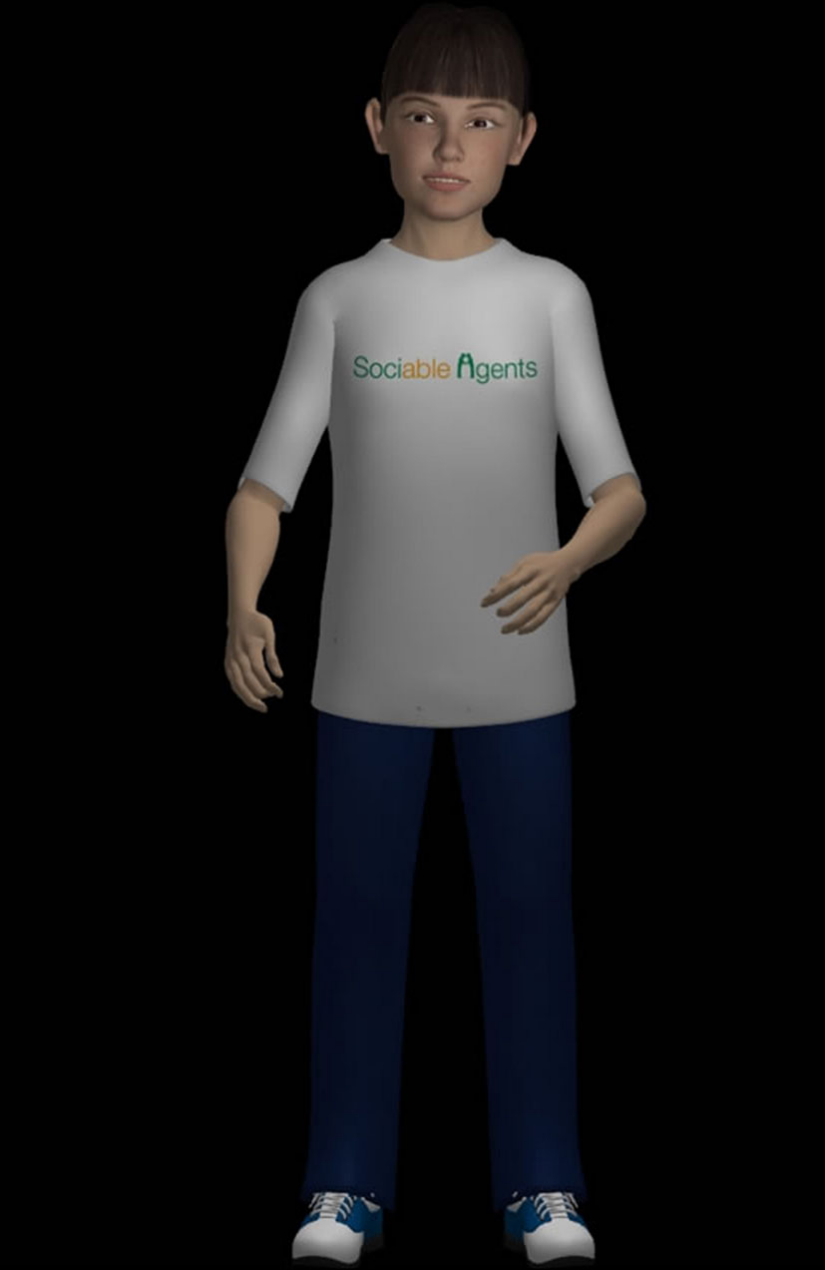
**Screenshot of the virtual agent Billie**.

In recent work we have focused on vocabulary learning as a first step toward foreign language acquisition (Bergmann and Macedonia, 2013). In our study, we have been employing the virtual human *Billie* (Figure 1), driven, technically speaking, by the AsapRealizer (Welbergen et al., 2012), which specifies the agent's behavior in Behavior Markup Language (BML) (Vilhjálmsson et al., 2007). BML coordinates speech, gesture, gaze, head and body movement. Thus, Billie, who looks like a young boy (thus pardon our personification), can show human-like behavior to a certain extent.

In his role as a vocabulary trainer, Billie enunciates words in a foreign language and at the same time performs iconic gestures representing the words (Bergmann et al., 2013). For example, for the word “book” his gesture simulates the opening of an imaginary book. Billie accompanies words with gestures because gestures enhance the retention of vocabulary. The enactment effect, i.e., the positive effect that gestures have on the memorization of verbal information, has been demonstrated in a number of experiments since the early eighties in research groups all over the world (see Zimmer, 2001, for a review). However, this knowledge has not been applied to foreign language vocabulary learning to any great extent. In the past decade only occasional studies have dealt with this topic (see Macedonia and Von Kriegstein, 2012, for a review). Educational practice still does not regard the body as a learning tool, although laboratory research has demonstrated that gestures support cognitive processes (Barsalou, 2008) and, in addition to language (Goldin-Meadow and Alibali, 2013), also enhance mathematical thinking and learning (Goldin-Meadow and LevineJacobs, 2014). This neglect of gestures persists despite the fact that second language practitioners have used self-performed pantomimes (Carels, 1981) and have been appraising the beneficial use of gestures in word learning since the eighteenth century (Radonvilliers, 1768).

In laboratory research, the enactment effect on memory has been explained in different terms, for example, as motoric imagery (Saltz and Donnenwerthnolan, 1981) or as a motor trace (Engelkamp and Zimmer, 1985) that complements the word's representation in memory and makes it resistant to decay (Klimesch, 1994). Most interestingly, neuroscience has shown that learning words through enactment leads to the formation of extended memory networks, including canonical language areas of the brain as well as several visual, sensorimotor, and associative areas involved in the encoding process (Masumoto et al., 2006; Macedonia et al., 2011). These extended networks account for short- and long-term memory enhancement compared to audio-visual learning (reading and hearing). Thus, empirical results regarding enactment have revealed that the body can successfully be used as a learning tool and that sensorimotor learning is a superior alternative to audio-visual learning (Macedonia, 2013). For these reasons, Billie was modeled to serve as an instructor to teach users new words by means of enactment.

## Can a virtual agent be a helpful teacher?

In order to assess whether an agent can train learners as well as a human teacher can, we conducted a within-subjects behavioral study (Bergmann and Macedonia, 2013) in which both a human trainer and a virtual agent trained 29 students. They learned 36 words in Vimmi, an artificial corpus that conforms to Italian phonotactics. Vimmi was constructed for experimental purposes in order to avoid associations with languages known to participants. We cued subjects to listen, read, and repeat the words and to watch videos in which the agent or the human trainer performed iconic gestures. Participants had to perform the gestures demonstrated by both trainers. The overall memory results reflected higher scores when participants learned with Billie; however, the difference was not significant. Because individual performance showed high variance among subjects, we used the median to split the population into high and low performers. Surprisingly, for high performers the agent-based training proved to be significantly more successful than the training with the human teacher. In order to explain this effect, we acquired data to determine how the agent is perceived as a trainer. Naive participants who had not trained with the agent previously were asked to rate the gestures and the “personality” of both the IVA and the human. Participants rated the human gestures as significantly better than those of the agent (more fluent, etc.). Interestingly, the perception of the “personalities” of the human and the trainer did not differ greatly. The only difference was that participants rated the human trainer as significantly more intelligent than Billie. We attributed the results to factors that we summarized as the “bizarreness” of the trainer (Macedonia and Bergmann, in press).

In another study, we tested Billie's performance as a virtual vocabulary trainer for 44 school children of mean age 12 (Macedonia et al., in preparation). In this experiment, children were trained in the classroom according to three conditions. Children listened to Vimmi words that were read to them along with their translation into German (condition 1); children watched semantically related, i.e., iconic gestures performed by the IVA (condition 2), or did both and imitated the gestures (condition 3). The overall results show that watching the agent while performing an iconic gesture significantly enhances word memorization compared to audio-visual learning. However, significantly better results were obtained when children imitated the agent, i.e., performed the gestures themselves.

In a further study (Macedonia et al., in preparation), we assessed the attitude of 12-year-old children toward IVAs. Similarly to the study with adults reported above (Macedonia and Bergmann, in press), this investigation was designed to determine how children perceived the gestures and the personality of the agent. Twenty-two school children age 11 were shown 15 gestures (videos) performed by both Billie and by a 12-year-old boy. The children were asked to rate the quality of the gestures and some of the personality traits (i.e., sympathy, friendliness, and intelligence) of both the agent and the child. The human gestures were rated as better than those produced by the IVA, as in the study with adults. However, the children did not perceive any significant difference in the sympathy and the intelligence of the human and the agent. Again, this behavioral study shows that children (at least this sample) also accept an IVA.

In summary, experiments conducted so far with the virtual agent Billie have demonstrated that he can train humans to learn vocabulary items as well as a human trainer. This is the case both for adults trained in a lab and for children trained in a classroom. In addition, we have shown that memory results improve if learners perform the gestures themselves instead of only watching the IVA perform them. Further, both young adults and children demonstrate good acceptance of the virtual trainer.

## Agents will become intelligent and serve as individualized personal trainers

In the experiments described above, the agent was not intelligent and did not interact with the users. The IVA did not provide feedback on gesture and pronunciation performance. However, as these experiments focused on learning with gestures, feedback would have represented an additional variable biasing the results. In fact, feedback does have an influence on motivation (Hattie, 2011; Busse, 2013) and consequently on learning. Recently this has also been demonstrated with respect to human/machine interaction. In a study by Mumm and Mutlu (2011), 192 participants were engaged in a speed-reading task; verbal feedback from the computer and the presence of a virtual agent on the screen positively influenced their task persistence. The authors conclude that both feedback and the agent enhanced motivation.

Because gesture performance leads to better results, participants must be instructed not only to perform the gestures but also to execute them accurately. We have observed (anecdotal evidence) that during training learners tend to reduce the gestures and/or omit them. In order to monitor learners, the agent must recognize motions performed by the user. Different technologies that enable recognition (Biswas and Basu, 2011; Ozcelik and Sengul, 2012) already exist and can be applied. The intelligent agent then compares the user's gestures with a template and allows a certain degree of deviation. If deviation surpasses a threshold, information is conveyed in spoken form, for example: “You did not move your right arm the way I told you to.” Monitoring each user's gestures ensures that learners enact the words in the most appropriate manner. This is necessary in order to create stable experience-dependent sensorimotor networks in their brains (Kiefer et al., 2007) that retain the foreign words. Furthermore, in order to train users to pronounce words like natives do, automatic speech-recognition software (ASR) can provide guidance. ASR systems detect differences in pronunciation from those in stored native speaker templates (Ma et al., 2012). If the deviation of the learner surpasses a threshold, the agent recognizes this and can trigger corrective feedback similar to the gesture correction. Corrective feedback from the agent involving both speech and facial expression animate the user to do better (Tung, 2011). ASRs are already in use, and their positive effects on motivation and achievement were recently reviewed (Golonka et al., 2012).

Another major issue concerning the development of IVAs is their customization to a user's special needs. During the experimental training described above, Billie taught participants without taking their intellectual capacities or their learning progress into account. The agent offered standardized training with a certain number of repetitions for all of the words. This training was inflexible and in a certain sense also inefficient. Some users might need more repetitions, while others might require fewer. It has been demonstrated that high performers who learn with gestures activate their brain resources differently than low performers do (Macedonia et al., 2010). This, in turn, leads to differences in learning achievement. Besides, some words might be easier for one person to learn than for another.

Hence, it is necessary to integrate all of this information into the training scheme provided to each individual user. An IVA will thus devise a standard cognitive profile, taking into account age, working memory performance, level of attention, education, and a few other parameters that are important in foreign language learning, such as cognitive control (Abutalebi et al., 2012) and bilingualism, as well as impeding factors such as dyslexia (Callens et al., 2012). The agent will then evaluate the frequency and duration of the training and match them with the learning results. Furthermore, the agent will calculate a standard deviation from the expected standard results for each particular learner. On this basis, the IVA will determine the amount of training (number of repetitions, frequency of training, etc.) that is necessary for any individual user. The longer the agent collects data on the user, the more finely the training can be tuned to individual needs. In this way, low and high performers can be challenged individually: frustration will be reduced but, most importantly, skills and capabilities will be enhanced.

Another aspect that needs to be implemented in the agent's interaction is personalized emotional supportive feedback. Whereas a human trainer can show differences in mood as well as sympathy or antipathy toward a person, an IVA will never do so. The agent's attitude toward the user will always be positive and appreciating and manifested by the absence of negative elements in communication. However, the agent will also be modeled to take the user's emotional state into account. Besides automatic speech recognition, new software enables an agent to detect changes in the pitch and tone of the voice that denote emotion (Ramakrishnan and Emary, 2013; Rao et al., 2013; Lech and He, 2014). Furthermore, empathy models that recognize negative emotional states in the user (Boukricha et al., 2013) will be implemented. This will enable the machine to generate adequate verbal support, so that the agent can interact with the user in a sensitive and personalized way.

## Agent application and future global contribution for society

Of course, IVAs will not be confined to desktops waiting for the user to come home and train. Instead, as applications they will accompany users in their mobile devices wherever they go. All the burdens connected with getting from home or from an office to classes, struggling through traffic jams and finding a parking space will be eliminated. Users will then rationally use their time to do what they need to do: learn the foreign language. IVAs will also fulfill their ultimate goal: to train the users at any time of the day or night, whenever they want to use them. A further advantage of IVAs will be their low cost. For the price of a fast food meal or probably even less, users from all social classes and with all levels of income will be able to enjoy personalized instruction designed according to neuroscientific findings and tailored to their individual cognitive capacities and needs.

The challenges for the future are manifold. First, every step in the development of IVAs must be validated with experiments reflecting the impact of agent-guided instruction on the user's cognitive performance. In other words, statistical evidence rather than descriptive theory must be the basis for pedagogical practice. Secondly, after vocabulary learning, syntax, and morphology will have to follow and be incorporated into the design of the language competence of the virtual trainer.

IVAs as language instructors are no longer a mere vision: in the past 10 years, basic research in cognition and neuroscience has paved a new avenue for instruction. Furthermore, artificial intelligence and technology have laid the foundations for novel applications in the interaction between humans and information systems. However, the work has been done in different fields of research. Presently we are connecting the dots, defining interfaces between disciplines, and creating interdisciplinary and international task forces to enable researchers with different backgrounds and skills to contribute to the development of IVAs that are capable of serving as foreign language instructors.

### Conflict of interest statement

The authors declare that the research was conducted in the absence of any commercial or financial relationships that could be construed as a potential conflict of interest.
